# Alterations in intermuscular coordination underlying isokinetic exercise after a stroke and their implications on neurorehabilitation

**DOI:** 10.1186/s12984-021-00900-9

**Published:** 2021-07-03

**Authors:** Jeong-Ho Park, Joon-Ho Shin, Hangil Lee, Jinsook Roh, Hyung-Soon Park

**Affiliations:** 1 Department of Mechanical Engineering, Korea Advanced Institute of Science and Technology, Daejeon, 34141 South Korea; 2 Department of Neurorehabilitation, National Rehabilitation Center, Seoul, 01022 South Korea; 3 Department of Biomedical Engineering, University of Houston, Houston, TX 77004 USA

**Keywords:** Stroke, Intermuscular coordination, Isokinetic movement, Biomechanical task constraints, Rehabilitation

## Abstract

**Background:**

Abnormal intermuscular coordination limits the motor capability of stroke-affected upper limbs. By evaluating the intermuscular coordination in the affected limb under various biomechanical task constraints, the impact of a stroke on motor control can be analyzed and intermuscular coordination-based rehabilitation strategies can be developed. In this study, we investigated upper limb intermuscular coordination after a stroke during isokinetic movements.

**Methods:**

Sixteen chronic stroke survivors and eight neurologically intact individuals were recruited. End-point forces and electromyographic activities of the shoulder and elbow muscles were measured while the participants performed isokinetic upper limb movements in a three-dimensional space. Intermuscular coordination of the stroke survivors and the control participants was quantified in the form of muscle synergies. Then, we compared the number, composition, and activation coefficients of muscle synergies and the end-point force between the groups. The correlation between the alteration of muscle synergies and the level of motor impairment was investigated.

**Results:**

Four and five muscle synergies in the stroke and control groups were observed, respectively. The composition of muscle synergies was comparable between the groups, except that the three heads of the deltoid muscle were co-activated and formed one synergy in the stroke group, whereas those muscles formed two synergies in the control group. When the number of muscle synergies between the groups matched, the comparable composition of muscle synergies was observed in both groups. Alternatively, the modulation of synergy activation coefficients was altered after a stroke. The severity of motor impairments was negatively correlated with the similarity of the post-stroke synergies with respect to the mean control synergies.

**Conclusions:**

Stroke-affected upper limbs seemed to modularize the activation of the shoulder and elbow muscles in a fairly similar way to that of neurologically intact individuals during isokinetic movements. Compared with free (i.e., unconstrained) movement, exercise under biomechanical constraints including the isokinetic constraint might promote the activation of muscle synergies independently in stroke survivors. We postulated the effect of biomechanical constraints on the intermuscular coordination and suggested a possible intermuscular coordination-based rehabilitation protocol that provides the biomechanical constraint appropriate to a trainee throughout the progress of rehabilitation.

## Background

Motor impairments following a stroke affect the daily lives, social participation activities, and the quality of life of stroke survivors. In addition to spasticity and muscle weakness, abnormal and stereotypical intermuscular coordination contributes to the limited motor capability of the stroke-affected limb. In the case of upper limbs, two classical patterns of abnormal intermuscular coordination are the coupling of shoulder abduction and elbow flexion (termed 'flexor synergy’) and the coupling of shoulder adduction and elbow extension (called 'extensor synergy’) [1, 2]. Previous studies have reported that abnormal intermuscular coordination produces the reduction of reaching distance and the horizontal work area of the hand [3–5] as well as the torque coupling between upper limb joints [3, 6].

Intermuscular coordination has been quantitatively evaluated in the form of muscle synergies for the last few decades. According to the muscle synergy hypothesis, the human body simplifies intermuscular coordination to manipulate the numerous degrees of freedom in the musculoskeletal system by modulating the activation of a limited number of muscle synergies (i.e., consistent co-activation patterns of muscle groups) rather than by controlling individual muscles. The information obtained from muscle synergy analysis includes the number, composition, and activation coefficients of muscle synergies. The number of muscle synergies indicates the complexity of intermuscular coordination. The composition of muscle synergies quantifies the muscles that are co-activated to achieve specific motor goals required for the given motor tasks, whereas the activation coefficient indicates the timing and activation magnitude of the synergies. From the perspective of data analysis, note that the muscle synergy patterns can be affected by biomechanical task constraints, selected muscles, and specification of electromyographic (EMG) data processes such as filtering and scaling [7–9].

How a stroke alters muscle synergies under varying biomechanical task constraints must be investigated to understand the impact of a stroke on neuromuscular control because intermuscular coordination can be affected by task constraints. Various motor tasks can be applied for upper limb rehabilitation depending on whether the training is focused on restoring the movement pattern or strength and on the external constraints applied to the trainee. We define an external constraint as the physical interaction between the trainee and the rehabilitative device, which is designed to provide an assistive or resistive force to or restrict the movement of the trainee. If no contact exists between the trainee and the device (i.e., a task has to be performed in free space) or if the device is compliant enough to ignore the interaction, then the task can be considered as unconstrained. Biomechanical task constraints used for upper limb rehabilitation can be categorized roughly as follows: unconstrained movement in free space, constrained movement assisted or resisted by a mechanical device, static (isometric) strengthening, and dynamic strengthening during movement. These task constraints can also be adopted for the training of intermuscular coordination; therefore, investigation of intermuscular coordination across various motor tasks is also important for developing effective training strategies for intermuscular coordination.

Different mechanisms of post-stroke changes in intermuscular coordination underlying shoulder and elbow joints have been reported for the following task constraints: free movement [10, 11], constrained (particularly, assisted) movement [12–14], and static strengthening [15, 16] (see Table 1). When stroke survivors performed free movements with their affected upper limbs, a higher number of muscles were activated together as a synergistic muscle group compared with the muscles of the unaffected limbs [10, 11]. The increased coupling between muscles was interpreted as the ‘merging’ of the two or more intact muscle synergies, and the extent of merging was proportionally correlated with the level of motor impairment [10]. In addition, merging could reduce the number of muscle synergies (i.e., reduce the complexity of intermuscular coordination) from five to one [11]. Furthermore, the complexity of intermuscular coordination was also reduced in free gait [17] and static strengthening using the hand and wrist at unconstrained (i.e., self-selected) postures [18]. In contrast, when static strengthening was performed using the shoulder and elbow under postural constraint (i.e., controlled upper limb posture), intermuscular coordination of neurologically intact controls and three-stroke groups with varying severity of motor impairment (i.e., mild, moderate, and severe) was explained with the same number of muscle synergies [15, 16]. While the composition of the synergies was comparable across all groups in general, a stroke induced changes in the composition of shoulder-related synergies mainly because of the co-activation of the three heads of the deltoid muscle. The prevalence of altered shoulder-related synergies increased under isometric conditions as the severity of motor impairment increased after a stroke.

**Table 1:** Previous studies on intermuscular coordination of the upper limb after a stroke

Task constraints	Study	Design
Free (unconstrained) movement	Cheung et al. (2012) [10]	Chronic stroke Seven virtual reality tasks or ballistic movements toward 12 targets in the 3-D space Affected limb (N = 31; FMA-UE: 0 ~ 66/66) vs*.* Unaffected limb
	Garcia-Cossio et al. (2014) [11]	Chronic stroke Six arm and hand movements Affected limb (N = 33; FMA-Hand: 0 ~ 11/24; Ashworth: 0 ~ 29/56) vs. Unaffected limb
Assisted (constrained) movement	Tropea et al. (2013) [12]	Subacute stroke Horizontal movement toward eight targets assisted by a therapeutic robot Stroke survivor (N = 6; FMA-UE: 8 ~ 36/66) vs. The neurologically intact (N = 10)
	Scano et al. (2018) [13]	Chronic stroke Hand-to-mouth movement in 3-D space assisted by a therapeutic robot Affected limb without assist (N = 22; FMA-UE: 12 ~ 64/66) vs. Affected limb with assist
	Runnalls et al. (2019) [14]	Chronic stroke Reaching toward 14 targets in 3-D space with varying level of weight support Stroke survivor (mild impairment; N = 7; FMA-UE: 56 ~ 66/66) vs. Stroke survivor (moderate-severe impairment; N = 6; FMA-UE: 9 ~ 45/66) vs. The neurologically intact (N = 6)
Static (isometric) strengthening	Roh et al. (2013, 2015) [15, 16]	Chronic stroke End-point force generation along 54 directions in the 3-D space at fixed posture (by holding the stationary force sensing handle) Stroke survivor (mild impairment; N = 8; FMA-UE: 50 ~ 66/66) vs. Stroke survivor (moderate impairment; N = 8; FMA-UE: 29 ~ 45/66) vs. Stroke survivor (severe impairment; N = 8; FMA-UE: 12 ~ 23/66) vs. The neurologically intact (N = 8)

Previous studies focusing on post-stroke patients suggest that the characteristics of intermuscular coordination, underlying the movement performed in support of external devices, can be different from those evaluated in free space after a stroke. The number and composition of muscle synergies during assisted horizontal or three-dimensional reaching tasks were comparable between stroke survivors and neurologically intact individuals; however, increased coupling of the deltoid muscles affected the composition of the shoulder-related synergies in the stroke group [12, 14]. When the three-dimensional reaching task of the affected limb was supported by weight supporters or therapeutic robots, the expression of the abnormal synergistic pattern of the shoulder muscles including deltoid muscles was reduced compared to when no assistance was provided [13, 14]. The motor deficit during the assisted reaching task after a stroke was mainly caused by the alteration of the activation coefficients of muscle synergies instead of the number and the composition of muscle synergies [12].

While previous studies have addressed upper limb intermuscular coordination that underlies free, constrained, and supported movement and static strengthening after a stroke, to the best of our knowledge, upper limb intermuscular coordination of dynamic strengthening tasks post stroke has not been investigated thus far. Isokinetic movement, which is a type of dynamic strengthening task, has been adopted for the restoration of motor capability as well as evaluation for stroke rehabilitation [19, 20]. In addition, few previous studies reported that isokinetic exercise improved upper limb motor function rated by the Fugl-Meyer assessment or box and block test and kinematic characteristics such as movement time and peak velocity [21, 22]. Thus, isokinetic movement has potential advantages during training for intermuscular coordination. Exercise at a controlled speed within the limited range of motion can prevent secondary muscle injury. In addition, it was reported that isokinetic movement promotes the activation of agonist muscles without provoking co-contraction of antagonist muscles [23]. Thus, the characterization of intermuscular coordination during isokinetic movements can provide a scientific foundation to design intermuscular coordination-based training strategies to improve the motor function of stroke survivors.

In our current study, we investigated the characteristics of upper limb intermuscular coordination of chronic stroke survivors during three-dimensional linear isokinetic movements. In addition to differences in the isokinetic muscle synergies between stroke survivors and neurologically intact people, we also evaluated how the characteristics of the stroke-affected isokinetic muscle synergies (such as the number of muscle synergies, synergy composition and activation profile after stroke with respect to those of control synergies) varies according to the level of motor impairment. Constraining the trajectory of the end-point of the upper limb on linear paths allowed stroke participants and healthy controls to achieve comparable end-point trajectories, while variations in joint kinematics caused by the differences in intermuscular coordination were still expressed. We expected that matching of end-point trajectories between the stroke and control groups would reduce undesired variations of the intermuscular coordination caused by differences in motion. By comparing the findings of this study with those of previous literature, we discussed how intermuscular coordination was affected by a stroke under various external biomechanical constraints. We also addressed how isokinetic task constraints can be adopted for training of intermuscular coordination in the stroke-affected upper limb. Specifically, stroke survivors and neurologically intact controls were recruited to perform eight upper limb movements in the three-dimensional space. Their muscle synergies underlying the activities of shoulder and elbow muscles as well as the end-point forces were analyzed. Correlations between alterations of muscle synergies and the level of motor impairment were examined.

## Methods

### Participants

Sixteen participants with chronic stroke (14 men and 2 women; age: 51.6 ± 7.9 years, range 30–62 years; months after a stroke: 56.8 ± 45.2 months, range 11–152 months) were recruited (Table 2). The inclusion criteria were as follows: (1) age range: 20 to 65 years, (2) upper limb hemiplegia without other neurologic and orthopedic diseases, (3) cognitive capability confirmed by a three-step command test, and (4) upper limb mobility to perform initial postures of the experimental tasks (shoulder flexion of approximately 45°, and shoulder abduction and elbow extension of 40°). Majority of the stroke participants were male, but it was shown that physical characteristics, motor performance and muscle synergy after stroke were sex-independent [15, 16, 24, 25]. Most stroke participants were middle-aged (i.e., 40–65 years) adults by reflecting increased prevalence of stroke among people over 40 years. To evaluate the effects of the level of motor impairment on the alteration in muscle synergies after stroke, participants with a wide range of scores of Fugl-Meyer assessment for upper limb (FMA-UE) were recruited. As the control group, eight neurologically intact people (3 men and 5 women; age: 54.3 ± 6.1 years, range 42–62 years) were recruited who had no orthopedic issues of the upper limb. To match the ages of the stroke and control participants, middle-aged people were recruited. This study was approved by the institutional review boards of the Korea Advanced Institute of Science and Technology as well as the National Rehabilitation Center of South Korea. All subjects provided written informed consent before the study.

**Table 2:** Participant demography

Stroke (N = 16)							Control (N = 8)			
Partici- pants	Sex (M/F)	Age (yrs)	Months after stroke	Affected Brain Side	FMA -UE	Removed EMGs	Partici-pants	Sex (M/F)	Age (yrs)	Removed EMGs
S01	M	60	34	Rt	63	SS	C01	M	54	
S02	M	50	91	Lt	43		C02	F	53	SS
S03	M	52	80	Lt	31	LT	C03	F	57	Brc
S04	M	47	27	Rt	49	Brd	C04	M	60	SS
S05	M	61	94	Rt	60	IS	C05	F	62	
S06	F	53	48	Rt	64		C06	F	54	
S07	M	54	152	Lt	17		C07	F	42	
S08	M	55	149	Lt	57		C08	M	52	
S09	M	30	25	Lt	22	IS				
S10	M	55	18	Rt	41	SS				
S11	M	51	12	Lt	26					
S12	F	53	11	Lt	54	Tlo, IS				
S13	M	53	47	Lt	29					
S14	M	40	38	Rt	37					
S15	M	50	64	Rt	12					
S16	M	62	19	Rt	37	Brc				
Mean	M 14,	51.6	56.8	Rt 8,	40.1		Mean	M 3,	54.3	
(SD)	F 2	(7.9)	(45.2)	Lt 8	(16.6)		(SD)	F 5	(6.1)	

*M* male, *F* female, *Rt* right hemiplegia, *Lt* left hemiplegia, *FMA-UE* Fugl-Meyer assessment for upper extremity (out of 66), *SS* supraspinatus, *LT* lower trapezius, *Brd* brachioradialis, *IS* infraspinatus, *Tlo* long head of triceps, *Brc* brachialis

### Experimental protocol

**Fig. 1:**
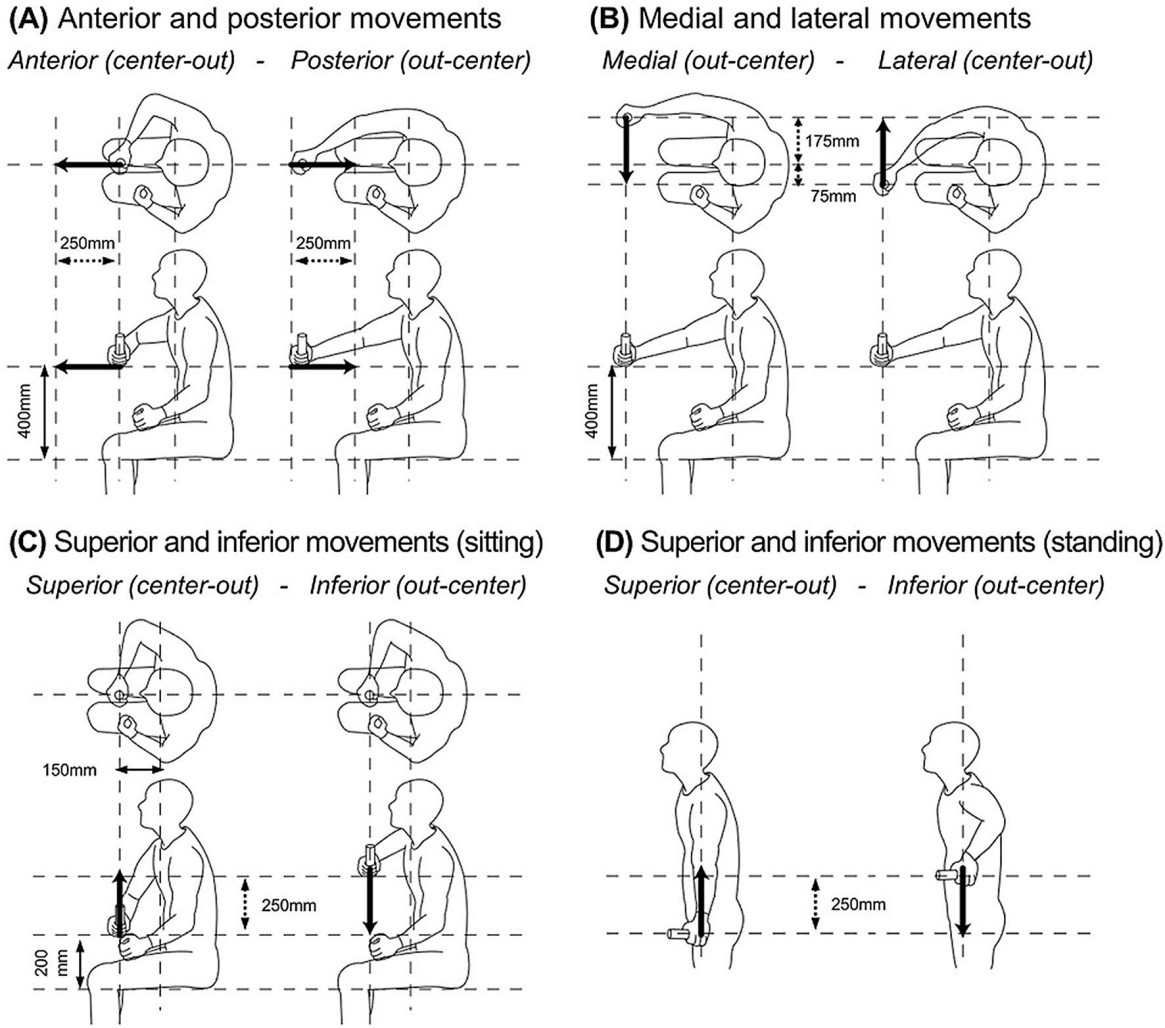
Upper limb movements examined in this study. The participants moved their hands 250 mm (see black arrows) in one direction and returned to the initial position after a rest of two seconds. The numbers with the black double-headed arrows represent key dimensions to determine the initial positions of the handle relative to the participants

**Fig. 2:**
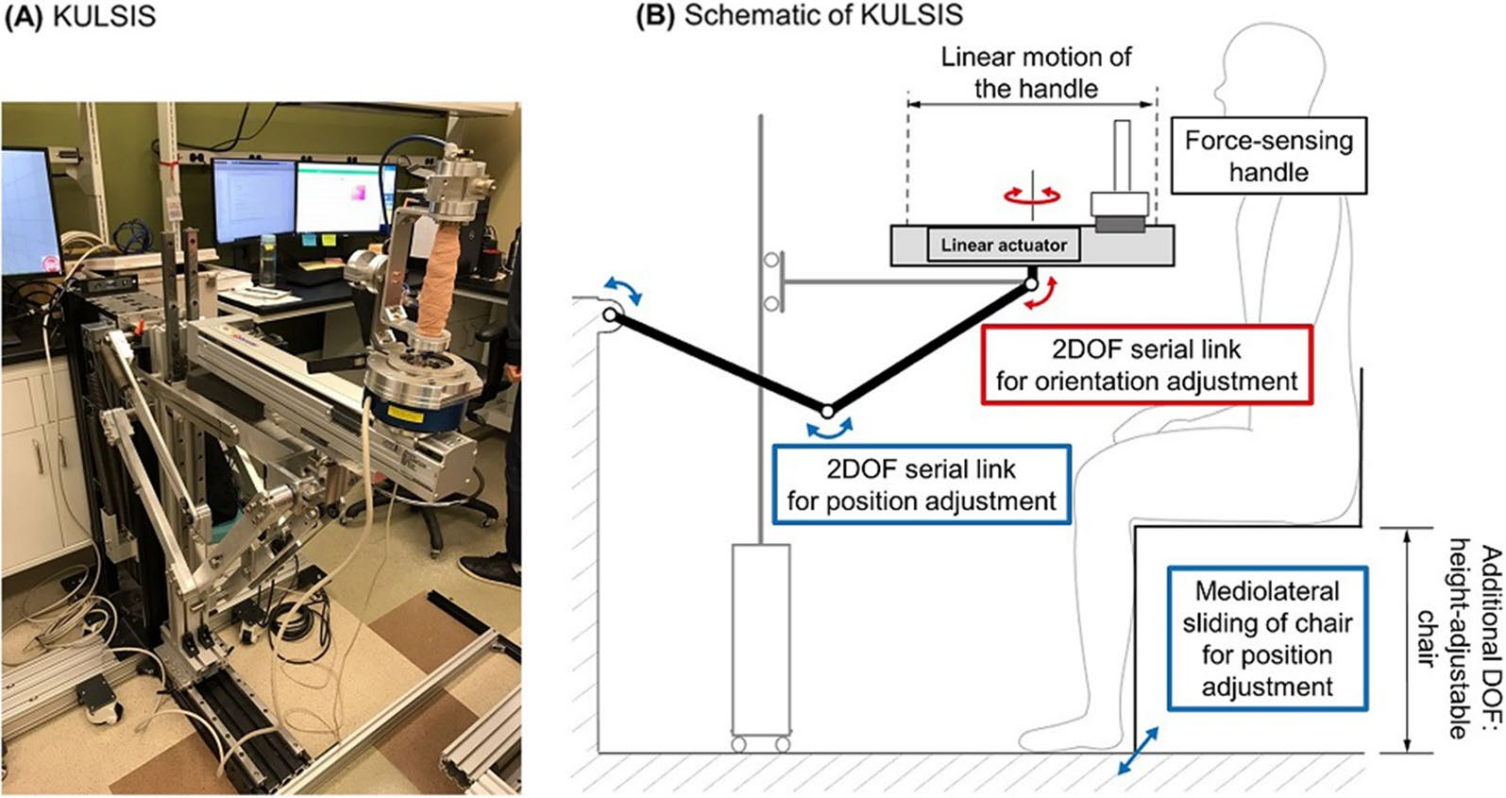
**A** Experimental setup, KULSIS, and **B** its schematic. The linear actuator is aligned to a participant using the 3DOF mechanism for position adjustment (blue arrows) and the 2DOF mechanism to adjust its orientation (red arrows)

**Fig. 3:**
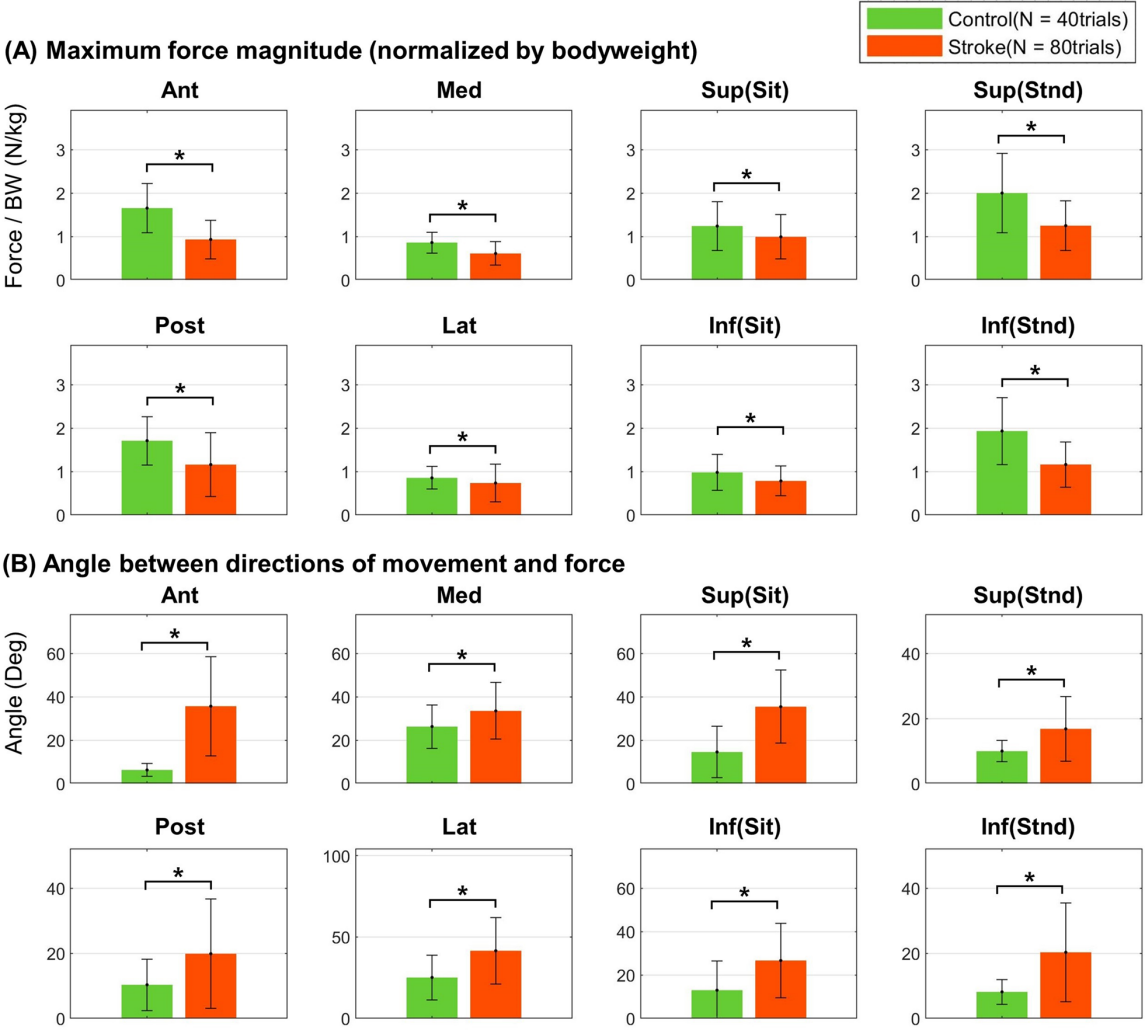
Inter-group difference of end-point force. **A** The mean maximum force magnitude normalized by bodyweight (BW), and **B** the mean angle between the directions of the movement and the force. The error bars represent one standard deviation. Asterisks represent the statistical difference between control and stroke groups (Wilcoxon rank-sum test, *p* < 0.050). *Ant* anterior movement, *Post* posterior movement, *Med* medial movement, *Lat* lateral movement, *Sup* superior movement, *Inf* inferior movement, *Sit* in a sitting posture, *Stnd* in a standing posture

The participants were asked to perform two isokinetic upper limb movements (i.e., center-out and out-center) for each of the following combinations of directions and postures: (1) anterior and posterior movements (Fig. 1A), (2) medial and lateral movements (Fig. 1B), and (3) superior and inferior movements (Fig. 1C), which were performed in the sitting posture, whereas (4) additional superior and inferior movements (Fig. 1D) were tested in the standing posture. Each movement was consecutively performed for five trials. Each trial comprised four steps: a 2 s baseline period (to measure baseline noise of EMG and force offsets), center-out and out-center periods (the participants were asked to move their hands 250 mm forward, medially or upward, and then to the initial location), and a 2 s ‘rest’ period between the center-out and out-center periods. Participants were asked to maximize the force generated along the direction of movement and to minimize the force generated along the other directions. If the participant was unable to move his hand by 250 mm, the distance was adjusted.

### Experimental setup

Ten surface EMG electrodes (Delsys Trigno EMG, Delsys, MA, USA) were employed to record EMG signals from the following muscles: upper trapezius (UT); lower trapezius (LT); clavicular head of pectoralis major (PM); anterior, middle, and posterior heads of the deltoid (AD, MD, and PD, respectively); long and lateral heads of triceps (Tlo and Tla, respectively); biceps (Bic); and brachioradialis (Brd). Surface EMG signals were recorded at 1200 Hz. In addition, three fine wire electrodes (Disposable Hook-Wire Electrode, Natus, CA, USA) were employed to record EMG signals from three deep muscles; supraspinatus (SS), infraspinatus (IS), and brachialis (Brc). An experienced physician placed the wire electrodes under ultrasonic guidance (Sonon, Healcerion, Korea). The intramuscular EMG signals were amplified using commercial differential amplifiers (INA-128, Texas Instrument, TX, USA; amplification gain, 1000; common-mode rejection ratio, 120 dB; and bandwidth, 4000 Hz) and were recorded at 4380 Hz.

The isokinetic upper limb movements along straight paths were implemented using an experimental device called the KAIST upper limb synergy investigation system (KULSIS) [26], which includes a linear actuator (RS-075 N-Z05PR, Robostar, Korea) that transports the handle linearly and a five-degree-of-freedom (DOF) mechanism to adjust the position and orientation of the linear actuator (Fig. 2). The three-dimensional force applied by the participants to the handle was measured using a force/torque sensor (Delta SI-660-60, ATI Industrial Automation, NC, USA), which connected the sliding block of the linear actuator and the handle. The handle was programmed to move at 20 mm/s back and forth when the participant applied a force larger than 5 N along the direction of the movement, which was easily achieved by participants with various levels of motor impairment. If the force component along the direction of movement was smaller than 5 N, the speed of the handle was proportional to the magnitude of the force. The EMG and end-point force data were collected by custom programs developed using the LabVIEW software (LabVIEW 2016, National Instruments, TX, USA).

### Signal processing

Prior to the introduction of data processing, all procedures were conducted using custom programs developed based on MATLAB software (MATLAB 2018a, MathWorks Inc., MA, USA). The surface and intramuscular EMG data were processed in the following sequence: (1) band-rejection filter (cut-off: 55–65 Hz), (2) low-pass filter (cut-off: 450 Hz for surface EMG, 1500 Hz for intramuscular EMG), (3) high-pass filter (cut-off: 30 Hz), (4) subtraction of the median for removing the DC offset, (5) rectification, and (6) low-pass filter (cut-off: 1 Hz) to obtain the envelope of the EMG signal. Then, the mean baseline EMG was subtracted from the processed EMG data to remove any tonic EMG signals, which were not task-dependent. Based on the end-point force and the handle speed, the EMGs of the three periods (i.e., center-out, out-center, and rest) were segmented. The segmented EMGs were resampled for a uniform length of 630 samples per trial (i.e., 300 samples each for the center-out and out-center periods and 30 samples for the rest period). If EMG data were contaminated by bad contact or severe noise in some trials, the data were excluded from further analysis and replaced with the average EMGs of the remaining trials for the same movement to ensure a uniform number of trials for all participants. If some EMG channels were contaminated in all trials for at least one movement, up to two EMG channels were excluded from further analysis (Table 1). Finally, the resampled EMGs of 20 trials were concatenated per participant and normalized by the maximum EMG amplitude of each muscle.

The three-dimensional end-point force of upper limb, which was applied to the handle of KULSIS by the participants, were collected at 1000 Hz. The raw force data were low-pass filtered (cut-off: 5 Hz). Note that we collected net forces after subtracting the force offsets which were measured during the baseline period. The filtered force data were also resampled in the same manner as the EMG data to match the length of data across trials. The positive and negative forces over a single axis of the force sensor were treated as two force components with opposite directions. The force data was normalized by bodyweight of each participant. For trials in which EMGs were contaminated, the corresponding force data were excluded from further analysis and replaced with the mean force data averaged over the remaining trials of the same movement. Per movement, the magnitude and direction of the three-dimensional end-point force were compared between the groups in order to evaluate how accurately the stroke survivors could control the force. The force magnitude was quantified as the maximum magnitude of the end-point force, the maximum value of the norm of the three-dimensional force vector during the movement. The force direction was quantified as the angle between the directions of the movement and of the force vector at the moment of the maximum force magnitude. The normality of the maximum force magnitude and the angle between the movement and force were tested for every movement (Chi-square goodness of fitness test; statistical significance, 0.05; control, N = 40 trials, 5 bins; stroke, N = 80 trials, 10 bins). In both cases of the maximum force magnitude and the angle between the movement and force, the normality was not proven in both groups for most movements. Thus, a nonparametric test was applied (Wilcoxon rank-sum test, statistical significance: 0.05).

### Identification of muscle synergy

We employed a non-negative matrix factorization (NNMF) algorithm [27] to decompose the concatenated EMG data into two components: composition of the synergies (W, synergy matrix) and their corresponding activation coefficients (C).

$${\text{EMG}}_{{{\text{126}}00{\text{X13}}}} = {\text{ C}}_{{{\text{126}}00{\text{XNsyn}}}} \cdot {\text{W}}_{{{\text{NsynX13}}}} + {\text{ R}}_{{{\text{126}}00{\text{X13}}}}$$

where N_syn_ is the number of muscle synergies and R is the residual EMG (i.e., unexplained variations in the given EMG). Each row of W represents each synergy vector (of unit magnitude) that specifies the balance of the 13 muscle weights. Each column of C represents the activation coefficient of each synergy vector at a given time. The variance accounted for (VAF), which is the squared sum of the residual EMG divided by that of the given EMG, quantifies the extent to which synergies could explain the total variance of the given EMG.

To determine the appropriate number of synergies, NNMF analysis was repeated by increasing the number of synergies from 1 to 10. For each synergy number, NNMF analysis was performed 25 times, and the set of synergies with the highest global VAF (gVAF; VAF obtained across all EMG channels) was chosen for further analyses. The appropriate number of synergies was estimated based on two criteria: (1) linearity of the gVAF curve with respect to the number of synergies [28, 29]; and (2) combination of several VAF metrics such as gVAF, VAF of each muscle (mVAF), and the increase in gVAF due to the increment of the number of synergies by one (∆gVAF) [15, 16, 18]. The first criterion was based on the fact that gVAF of random EMG data linearly increases as the number of synergies increases. This method sought the minimum number of synergies such that the gVAF curve after that number of synergies became linear (i.e., remaining variations of EMG data could be considered meaningless). In this study, the linearity was confirmed using the mean squared error of linear regression (MSE) below 10^–4^. The second criterion sought the minimum number of synergies to meet the threshold of all VAF metrics. For example, in this study, gVAF and mVAF should be larger than 0.9 and 0.7, respectively, whereas ∆gVAF should be smaller than 0.05. The appropriate number of synergies was identified for each participant’s data. The number of synergies that satisfied both criteria was chosen and averaged across participants in the same group to determine the mean number of synergies for each group. Then, the mean number of synergies was approximated to the nearest integer and applied to the group to identify the appropriate synergies for each participant of the group for ease of comparison. Because the average number of synergies of the stroke group was different from that of the control group (i.e., four synergies, stroke *vs* five synergies, control), the muscle synergies of the stroke participants were analyzed so that they had five synergies. Then, muscle synergies of the two groups were compared again under the condition of the matched complexity of intermuscular coordination.

The gVAF values of the stroke and control groups achieved by the same number of synergies were compared. Because of the limited numbers of the sample (control, N = 8 subjects; stroke, N = 16 subjects), a nonparametric test (Wilcoxon rank-sum test, statistical significance: 0.05) was conducted. In addition, the correlation coefficient between the appropriate number of synergies per participant of the stroke group and the FMA-UE score was obtained to verify the relation between the complexity of intermuscular coordination and level of motor impairment.

Muscle synergies of individual participants were ordered with respect to those of the template participant of the same group to maximize the total similarity among synergy vectors and activation coefficients. The similarity of synergy vectors was calculated as a sum of the scalar products of the corresponding synergy vectors of the two participants in comparison. For participants whose one or two EMG channels were removed during data processing, the same EMG channels were also removed from the synergy vectors of the template participant. Then, the template synergy vectors were normalized again to obtain a unit vector before calculating the similarity. The similarity in the activation coefficients was defined as the sum of the scalar product of activation coefficients of the corresponding synergies in comparison. Participants C08 and S08 were chosen as the template subjects for the control and stroke groups, respectively.

### Comparison of muscle synergy

The composition of muscle synergies of each stroke participant was compared with the mean pattern of the control participants. The similarities in the synergy composition were calculated as a scalar product between each synergy vector of the stroke participant and the corresponding mean synergy vector of the control group. In addition, the mean value of the similarity of the individual synergy vectors was calculated to measure the similarity of a set of synergy vectors. When the number of synergies of the stroke group was smaller than that of the control group, one synergy of the stroke group could be matched to multiple synergies of the control group. If so, the synergy pair that achieved the largest similarity value was considered to have matched synergies between the two groups**.** Correlation between the FMA-UE score and the similarity of the synergy vector sets was investigated.

The similarity values obtained for the 16 stroke participants were averaged and compared with the similarity by chance, which was obtained as follows. For the similarity between individual synergy vectors, 1000 random synergy vectors were generated by sampling the activation amplitude of each muscle at random instances from the normalized EMGs of each stroke survivor [30]. Another 1000 random synergy vectors were generated from normalized EMGs of each control participant. The scalar product of synergy vectors was calculated from the 1000 pairs of random synergy vectors of the stroke and control groups. For analysis of the similarity between the sets of synergy vectors, 1000 sets of four and five random synergy vectors were generated for each stroke participant. Another 1000 sets of five random synergy vectors were generated for each control participant. The similarity between the random synergy vector sets of each stroke and each control participant was obtained by ordering the synergy vector sets in comparison and calculating the average of the scalar products between the corresponding random synergy vectors. The 95th and 90th percentile (i.e., the top 5% and 10%, respectively) of the similarity among the 1000 pairs of individual synergy vectors or synergy vector sets were averaged across all combinations of the stroke and control participants. Thus, 0.767 (0.710) was the 95th (90th) percentile of the similarity by chance of individual synergy vectors. When the number of synergies of the stroke survivors was four and five, 0.703 (0.678) and 0.714 (0.694) were the 95th (90th) percentile of the similarity by chance of the synergy vector sets, respectively. In addition, to compare the synergy composition when the number of synergies in the stroke group was four and five, the four stroke synergies were represented as linear combinations of five stroke synergies. The coefficients of linear combination (i.e., merging coefficients) indicate the synergies that were merged when the number of synergies decreased from five to four in the stroke group. The merging coefficients were obtained per stroke participant and were averaged across them. In particular, non-negative least square optimization was performed to avoid negative merging coefficients, which do not make sense physiologically. Only a mean merging coefficient above 0.2 was considered to denote significant merging of synergies [10].

If the mean similarity of the synergy vector sets of the stroke and control groups was higher than the similarity by 95% chance, the compositions of the stroke and control synergies were considered to be similar, and their activation coefficients were compared. To minimize the potential bias due to differences in the synergy composition, five synergy vectors common to all stroke survivors and control participants were obtained from the EMG dataset in which EMGs of both groups were concatenated. Because the number of control participants was half that of the stroke participants, the EMGs of the control group were concatenated twice to match the number of samples of the control and stroke EMGs. Note that EMGs of the supraspinatus and infraspinatus muscles were removed from the concatenated EMG because both muscles were removed from more than three participants. If these muscles were included in the concatenated EMG, they were identified as a separate synergy vector. The activation coefficient corresponding to the common synergy vectors was averaged per trial. The normality of the mean activation coefficients was proven in both groups for only 10 cases among 40 combinations of movements and synergies (Chi-square goodness of fitness test; statistical significance, 0.05; control, N = 40 trials per movement, 5 bins; stroke, N = 80 trials, 10 bins). Thus, the activation coefficient was compared between stroke and control participants for each movement using a non-parametric test (Wilcoxon rank-sum test, statistical significance: 0.05). Note that the initial and final 15 samples (i.e., 5% of 300 samples of each trial) were excluded from the calculation of the mean activation coefficients. If the group mean of the activation coefficient was smaller than 0.1 in both groups or if the inter-group difference of the group mean of the activation coefficient was smaller than 0.1, the level of activation was considered to be similar. In addition, the similarity of the activation coefficients of each stroke participant with respect to the mean activation coefficient of the control group was evaluated. Then, the similarity of the activation coefficients of five synergies was averaged per participant, and its correlation with the FMA-UE score was investigated.

## Results

### Maximum magnitude and direction of the end-point force

The accuracy of the stroke survivors in generating end-point forces along the direction of movement with compounding and unintended directional forces was lower than that of the control participants (Fig. 3). In particular, the maximum force magnitudes of the stroke group were statistically smaller than those of the control group for all movements (Wilcoxon rank-sum test; control, N = 40 trials; stroke, N = 80 trials; anterior, posterior, medial, lateral, superior(standing) and inferior(standing): *p* < 0.010; superior(sitting): *p* = 0.015; inferior(sitting): *p* = 0.017). In addition, the direction of the end-point force generated by the stroke participants deviated more from the direction of movement compared with that of the control group for all movements (all movements: *p* < 0.010).

### Complexity of intermuscular coordination

**Fig. 4:**
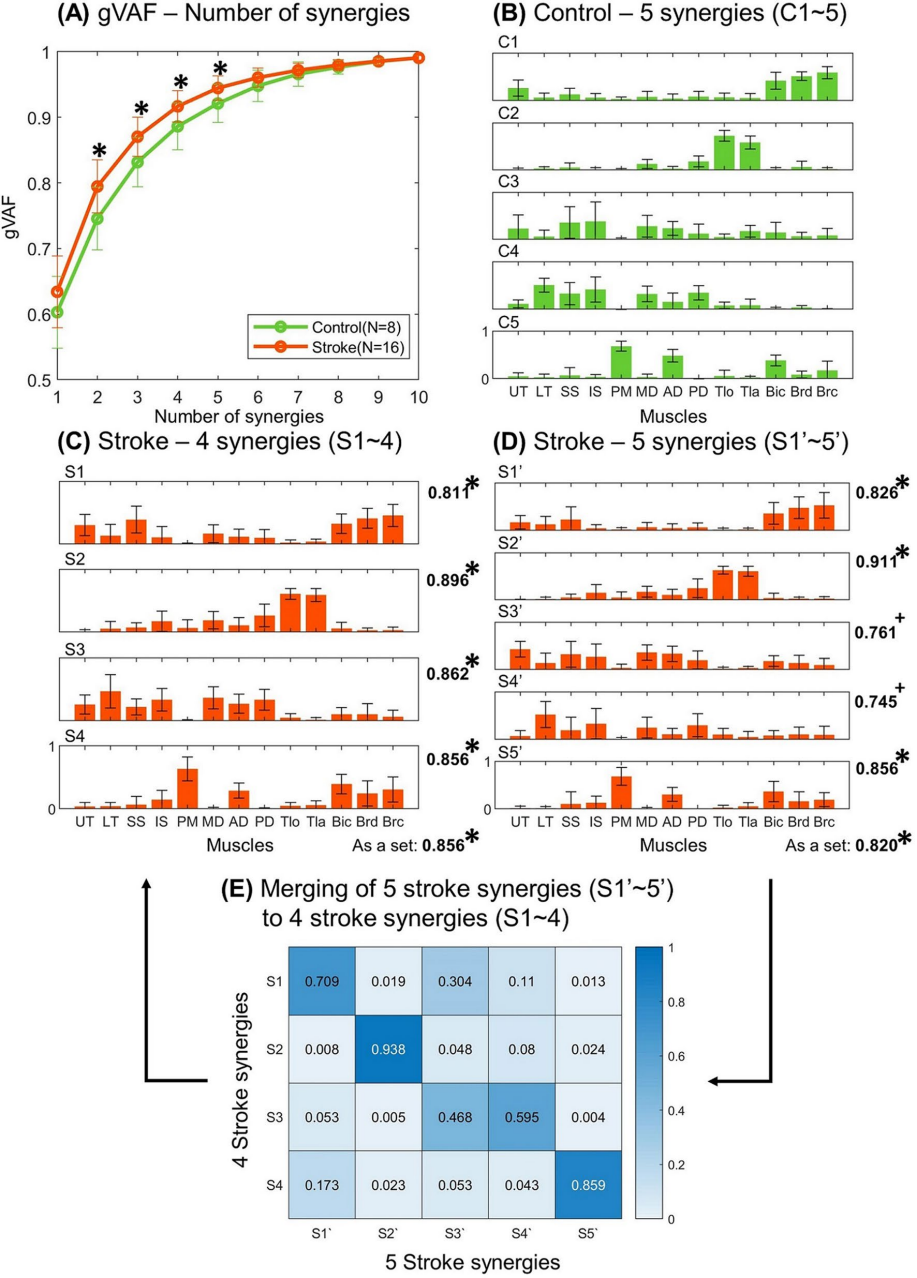
Number and composition of muscle synergies. **A** The gVAF values (mean ± SD) of the stroke (orange) and the control (green) groups. An asterisk represents a statistical difference between the two groups (Wilcoxon rank-sum test, *p* < 0.050). The mean composition of **B** five synergies (C1 ~ C5) of the control group, **C** four synergies (S1 ~ S4) of the stroke group, and **D** five synergies (S1' ~ S5') of the stroke group. Standard deviations are represented as error bars. The numbers to the right of the graphs represent the mean similarity of the individual synergy vector and the similarity of the set of synergy vectors of stroke participants to the mean synergy vectors of the control group. An asterisk represents a statistical difference between each similarity value and its corresponding similarity by chance (Sign test, *p* < 0.050). **E** Mean merging coefficients of five to four stroke synergy vectors. The merging coefficients above 0.2 are considered as a significant merging of synergies. *UT* upper trapezius, *LT* lower trapezius, *SS* supraspinatus, *IS* infraspinatus, *PM* pectoralis major, *MD* middle head of deltoid, *AD* anterior head of deltoid, *PD* posterior head of deltoid, *Tlo* long head of triceps, *Tla* lateral head of triceps, *Bic* biceps, *Brd* brachioradialis, *Brc* brachialis

EMGs of the stroke group were explained with a smaller number of synergies compared with that of the control group (Fig. 4A). In the range of the number of synergies from two to five, higher gVAF values (control: 0.745–0.921 vs. stroke: 0.795–0.945) were obtained for the stroke group (confirmed by Wilcoxon rank-sum test; control, N = 8 subjects; stroke, N = 16 subjects; *p* = 0.011–0.020). The control participants utilized 5.25 muscle synergies on an average, whereas the stroke survivors utilized 4.38 muscle synergies. The mean numbers of muscle synergies were statistically different between the groups (Wilcoxon rank-sum test; control, N = 8 subjects; stroke, N = 16 subjects; *p* = 0.028). In the subsequent analyses, the intermuscular coordination of the control and stroke groups was explained using five and four synergies, respectively. In addition, five synergies were identified from the EMGs of the stroke group to investigate inter-group differences in the synergy composition despite the matched complexity of intermuscular coordination.

### Composition of muscle synergies

The five synergies of the control group (C1–5 in Fig. 4B) were as follows: elbow flexor (UT, Bic, Brd, and Brc); elbow extensor (Tlo and Tla); shoulder flexor/abductor (UT, LT, SS, IS, MD, and AD); shoulder extensor/abductor (UT, LT, SS, IS, MD, and PD); and shoulder flexor/adductor (PM, AD, and Bic), respectively. When the four synergies were identified from the stroke participants (S1–4 in Fig. 4C), the first, second, and last stroke synergies (S1, 2, 4) were elbow flexor, elbow extensor, and shoulder flexor/adductor synergies, respectively. Interestingly, the third stroke synergy (S3) included the three heads of the deltoid muscle as well as the trapezius and rotator cuff muscles (i.e., supraspinatus and infraspinatus), whereas these muscles formed two separate synergies (C3 and C4) in the control group. The similarities in the four stroke synergy vectors and the corresponding mean synergy vectors of the control group were analyzed. The third stroke synergy was compared with both the third and fourth synergy vectors of the control group, and the higher value of similarity was chosen. Each of the four stroke synergy vectors had a similarity above the 95% chance with its corresponding control synergy vector (similarity by chance, 95% percentile = 0.767; S1 vs. C1: mean similarity = 0.811; S2 vs. C2: mean similarity = 0.896; S3 vs. C3 or C4: mean similarity = 0.862; S4 vs. C5: mean similarity = 0.856). The set of the four stroke synergy vectors had similarity above the 95% chance to the set of mean synergy vectors of the control group (similarity by chance, 95% percentile = 0.703; S1–4 vs. C1–5: mean similarity = 0.856).

If the intermuscular coordination of the stroke group was explained using five synergies (S1’–5’ in Fig. 4D), the three heads of the deltoid muscle were differentiated into two separate synergies (S3’ and S4’). Among the five stroke synergies, the similarities of the first, second, and fifth synergy vectors were larger than the similarity by 95% chance with their corresponding control synergy vectors (similarity by chance, 95% percentile = 0.767; S1’ vs. C1: mean similarity = 0.826; S2’ vs. C2: mean similarity = 0.911; S5’ vs. C5: mean similarity = 0.856). The similarity values of the third and fourth stroke synergies were lower than the 95% chance but were above the 90% chance (similarity by chance, 90% percentile = 0.710; S3’ vs. C3: mean similarity = 0.761; S4’ vs. C4: mean similarity = 0.745). As a set of synergy vectors, the five stroke synergy vectors had a similarity above the 95% chance (similarity by chance, 95% = 0.714; S1’–5’ vs. C1–5: mean similarity = 0.820).

**Fig. 5:**
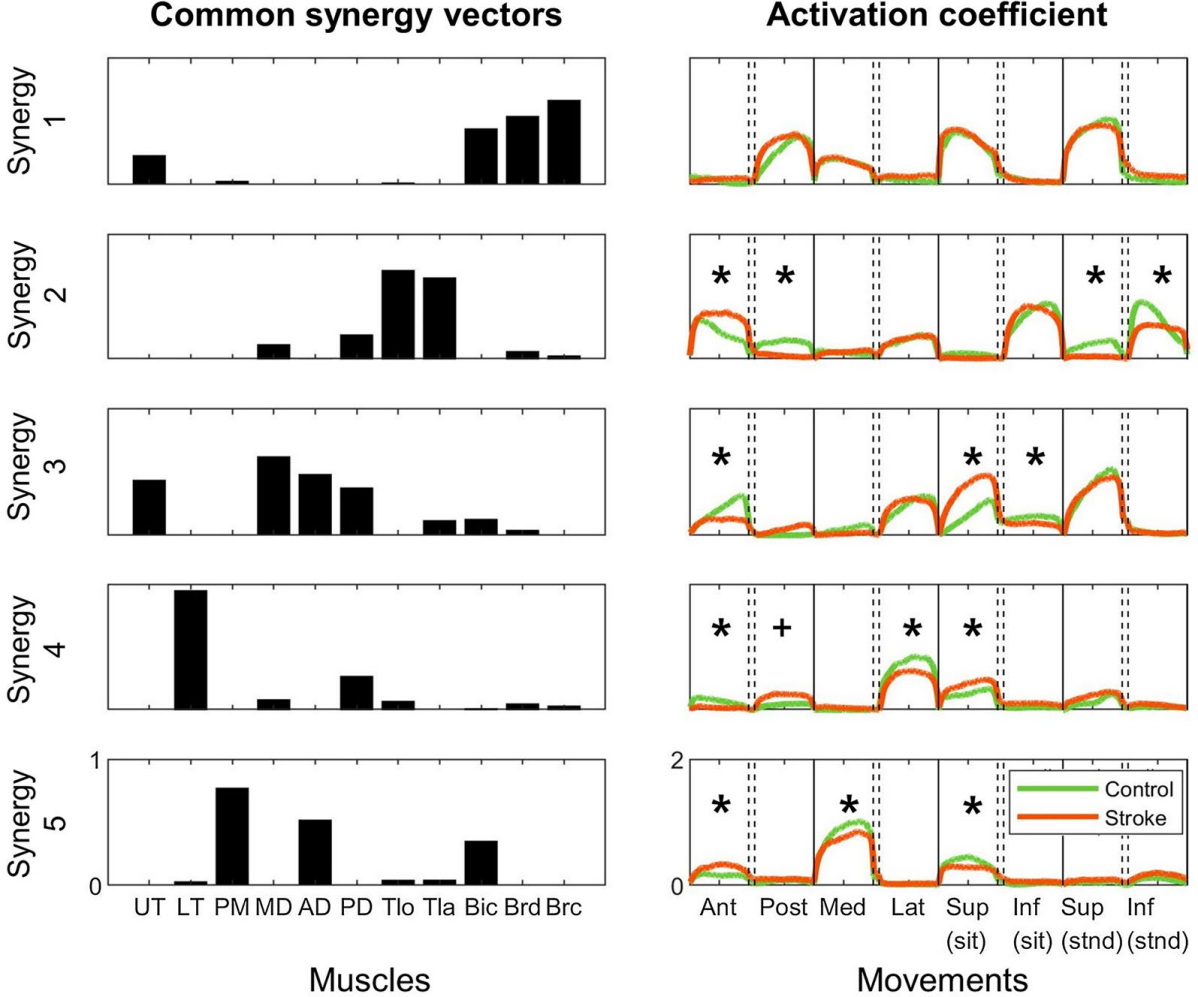
Common synergy vectors (left) and their activation coefficients (right). Activation coefficients of the common synergy vectors were compared between the stroke survivors (orange) and the control participants (green). Asterisks represent the statistically significant difference in the activation coefficient between the groups (Wilcoxon rank-sum test, *p* < 0.05). A cross implies the difference in which statistical significance was marginal (*p* = 0.05). *Ant* anterior movement, *Post* posterior movement, *Med* medial movement, *Lat* lateral movement, *Sup* superior movement, *Inf* inferior movement, *Sit* in the sitting posture, *Stnd* in the standing posture

When the number of the stroke synergies decreased from five to four, two cases of significant merging of synergies (i.e., merging coefficients above 0.2) were identified (Fig. 5). In the first case, the elbow flexor synergy (S1’) and the shoulder flexor/abductor synergy (S3’) were merged to form the elbow flexor synergy (S1; mean merging coefficient of S1' to S1: mean merging coefficient = 0.709; S3' to S1: mean merging coefficient = 0.304). In the second case, the shoulder flexor/abductor synergy (S3') and the shoulder extensor/abductor synergy (S4') were merged to form the shoulder abductor synergy (S3; S3' to S3: mean merging coefficient = 0.468; S4' to S3: mean merging coefficient = 0.595). In particular, the mean merging coefficients of the S3’ and S4’ to S3 were similar, implying that both S3’ and S4’ contributed to the formation of S3 to a similar extent.

### Activation coefficients of muscle synergies

Five muscle synergy vectors common to both stroke and control participants were identified to compare the activation coefficients among groups (Fig. 5). The gVAF achieved by the common synergy vectors was 0.904. In the control group, the first synergy, elbow flexor synergy, was used during the posterior and medial movements and superior movements in both the sitting and standing postures. In contrast, the second synergy, elbow extensor synergy, was activated when the participants moved their hand in the anterior, lateral, and inferior directions. The third and fourth synergies, which were composed of the shoulder flexor/abductor muscles and shoulder extensor/abductor muscles, respectively, were activated during the anterior, lateral, and superior movements in both postures. The fifth synergy, shoulder flexor/adductor synergy, was activated during the anterior, medial, and superior movements in the sitting posture.

Several alterations in the activation coefficients were observed between the stroke and control groups. For example, during anterior movement of the stroke participants, activation of the elbow extensor synergy increased (Wilcoxon rank-sum test; control, N = 40 trials; stroke, N = 80 trials; *p* < 0.010) and activation of the two shoulder abductor synergies decreased (*p* < 0.001 for both synergies) compared with those of the control group. In contrast, activation of the shoulder flexor/adductor synergy increased in the stroke group (*p* < 0.010). During the posterior movement of stroke participants, the shoulder extensor/abductor synergy was activated to a larger extent than that of the control group, and this difference was statistically marginal (*p* = 0.05). The shoulder flexor/adductor synergy was activated to a lesser extent in the stroke group when the participants performed medial movement than in the control group (*p* < 0.010). When the participants in the stroke group performed lateral movement, the activation of the shoulder extensor/abductor synergy was reduced (*p* < 0.010). The stroke survivors activated the shoulder abductor synergies more during the superior movement in the sitting posture (*p* < 0.010 for both synergies). The shoulder flexor/abductor synergy was activated to a lower extent in the stroke group during the inferior movements in the sitting posture (*p* < 0.010). In addition, the activation of the shoulder flexor/adduction synergy was decreased in the stroke group during the superior movement in the sitting posture (*p* < 0.010). When the stroke participants performed the superior and inferior movements in the standing posture, the activation of elbow extensor synergy increased in the control group (*p* < 0.010 for both movements), whereas the shoulder-related synergies showed no difference in activation coefficients between both groups.

### Correlation of alterations of intermuscular coordination and the level of motor impairments

**Fig. 6:**
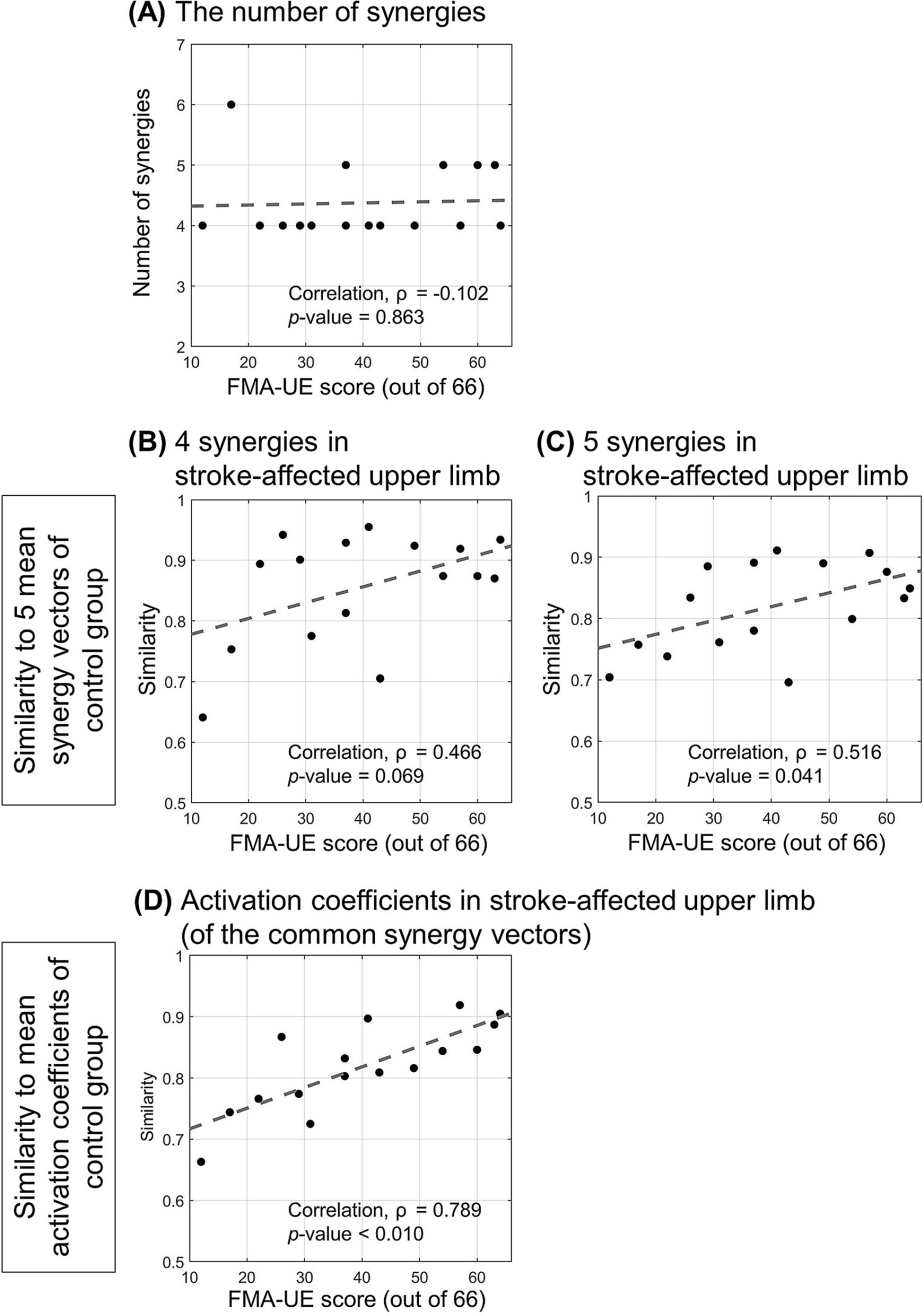
Correlation of muscle synergies post stroke and the FMA-UE score. **A** the number of synergies, the similarity scores of **B**, **C** synergy vector, and **D** activation coefficient of stroke participants with respect to mean synergy vectors and activation coefficient of the control group. The grey broken lines represent the lines of linear regression

The appropriate number of synergies identified per participant of the stroke group was not associated with the level of impairment (*p* = 0.863; Fig. 6A). As the level of motor impairment decreased (i.e., the FMA-UE score increase), the synergy vectors and the activation coefficients of the participants with a stroke tended to become more similar to the mean synergy vectors and the mean activation coefficients of the control group. The similarity of the synergy vectors was not correlated with the FMA-UE scores when the appropriate number of synergies of the stroke group was four (correlation coefficient, ρ = 0.466; *p* = 0.069; Fig. 6B) but the similarity of synergy vectors was correlated with the FMA-UE scores in the case of the five stroke synergies (ρ = 0.516; *p* = 0.041; Fig. 6C). The activation coefficients of the stroke survivors corresponding to the common synergy vectors were more dissimilar to those of the control groups as their level of motor impairment was more severe (ρ = 0.789; *p* < 0.010; Fig. 6D).

## Discussion

### Intermuscular coordination during isokinetic movement

The EMG underlying the isokinetic upper limb movements of the stroke survivors could be explained by a smaller number of muscle synergies than that of the neurologically intact group. This result implies that the stroke participants lacked the ability to activate the upper limb muscles in task-specific ways. However, the difference in the number of muscle synergies was relatively small (i.e., five in the control group vs. four in the stroke group) compared with that observed in the study of unconstrained tasks, such as free reaching, isometric hand tasks at self-selected gestures, and free gait [11, 17, 18]. The variation in the muscle activation of the stroke participants could be accounted for by four or five synergies, and the number of muscle synergies was not correlated with the level of motor impairment. This finding implies that the isokinetic constraints may reduce the extent of the merging of intact muscle synergies in the stroke population.

The major difference in the composition of muscle synergies between the stroke and control groups was the co-activation of the three deltoid muscles in the stroke group, even though the synergy composition was similar between the groups despite of the coupling. In our study, when the number of synergies was matched between the stroke and control groups, the deltoid muscles formed two separate synergies. When the number of synergies in the stroke group was four, the two deltoid-related synergies were merged into one synergy. The coupling of deltoid muscles was also observed during other mechanically constrained tasks [12, 14–16]. This abnormal coordination of the shoulder muscles induced abnormal shoulder abduction during isometric force generation [15]. Overall, these findings suggest a new intermuscular coordination-based rehabilitation strategy that aims to reduce the coupling of the deltoid muscles in the post-stroke upper limb.

To account for the difference in the end-point force between the stroke survivors and the control participants, we focused on another aspect of intermuscular coordination, the activation coefficient of muscle synergies. We observed that the activation of synergies was altered more (i.e., low similarity with respect to the mean activation coefficient of the control participants) in stroke survivors as the severity of motor impairment increased. Interestingly, the activation magnitude of the shoulder abductor synergies decreased and/or the activation of the shoulder adductor synergy increased in the stroke group when the stroke participants performed movements requiring elbow extension such as the anterior, lateral, and inferior movements. In contrast, the stroke group activated the shoulder abductor synergies more and/or activated the shoulder adductor synergy less than the control group during the posterior and superior movements, which required elbow flexion. These coupling patterns of the shoulder and elbow muscles coincide with the clinical observations on abnormal shoulder and elbow movements, flexor synergy and extensor synergy [1, 2] and the abnormal coupling of joint torques of shoulder and elbow in the stroke-affected upper limb [6]. The previous study on free gait in stroke-affected lower limbs showed the comparable composition of synergies between the stroke and the control groups when the number of stroke synergies were matched to that of control synergies [17]. Instead of synergy composition, the study concluded that a lack of independent activation of the synergies reduced the complexity of intermuscular coordination in the stroke participants of relatively poor gait performance. Similarly, in this study, the altered activation coefficients in the stroke group may have resulted in the deviation of their end-point force direction from the direction of the movement compared to that of the control group. Training of activation patterns of muscle synergies can be considered as a possible strategy for rehabilitation of abnormal intermuscular coordination after a stroke.

In summary, the stroke participants could have utilized generally comparable muscle synergies except the increased coupling of the deltoid muscles compared with those of the neurologically intact individuals during the isokinetic movements. The stroke group, however, activated their synergies differently than the control group and generated the end-point force less accurately. Thus, the isokinetic movement constraint can be utilized to train new activation profiles of the muscle synergies for improving the end-point force control during upper limb movements. Since it was also reported that the altered activation of lower limb muscle synergies could contribute to the reduced complexity of intermuscular coordination underlying free gait after stroke [17], this method might be applicable to restoring the stroke-affected intermuscular coordination for gait motions despite the difference in exercise condition. Considering the correlation between the complexity of intermuscular coordination and the gait quality measures in the stroke-affected lower limbs [31], gait performance might be improved by recovering the complexity of intermuscular coordination through the proposed training. In addition, a separate training regime to reduce the abnormal co-activation of the deltoid muscles can be accompanied by the training of new synergy activation profiles to rehabilitate stroke-affected intermuscular coordination of upper limb muscles effectively. We should emphasize that these proposed trainings of intermuscular coordination complement to conventional rehabilitation protocol of post-stroke motor impairment. The altered intermuscular coordination seems to be one of the factors which contribute to the inaccurate end-point force control in stroke-affected limbs. Therefore, it is necessary to evaluate the degree to which the accuracy of the force control can be improved through the proposed training.

### Consideration of task conditions for effective rehabilitation of intermuscular coordination

Task condition (i.e., its corresponding external constraint) is an important factor in rehabilitative training. In the context of intermuscular coordination, the task condition can affect the way in which a person achieves a given motor goal; thus, different intermuscular coordination can be observed. Recently, Pellegrino et al. evaluated variations in the upper limb intermuscular coordination in people with multiple sclerosis under four task conditions including free movement as well as different task conditions such as movements with assistive and resistive forces and static (isometric) strengthening [32]. They concluded that external constraints can affect intermuscular coordination and that it is necessary to provide appropriate task conditions for effective training of intermuscular coordination.

Unfortunately, no study has compared the characteristics of upper limb intermuscular coordination across varying biomechanical task conditions in patients with a stroke. Instead, intermuscular coordination has been evaluated for a wide range of tasks in various studies of heterogeneous design (Table 1) [10–16]. By comparing their findings, we discussed how the intermuscular coordination post stroke was affected by the presence of the task constraints. In the free space without external constraints, people with a stroke could perform the given tasks in their own preferred ways as a compensatory mechanism (i.e., at varying postures, using different joints to achieve a motor goal, etc.) significantly different from those of neurologically intact individuals. This difference in motor strategy could result in different (especially, simpler) intermuscular coordination patterns in stroke participants [11, 17, 18]. In contrast, if external constraints were provided, the difference in the complexity of upper limb intermuscular coordination was reduced between the stroke and control groups [12, 14–16]. In addition, the composition of most synergies was comparable in general although the composition of shoulder-related muscle synergies was still altered in the stroke group due to the co-activation of the shoulder muscles. Our result of intermuscular coordination underlying isokinetic movements was consistent with those of the previous studies that adopted the other constrained tasks.

To the best of our knowledge, biomechanical task constraints seem to be advantageous for the training of new activation profiles of muscle synergies because they promote stroke survivors to use their synergies in a more task-specific manner (i.e., higher complexity of intermuscular coordination). However, it is still unclear whether the different types of external constraints such as isometric strengthening or assisted movements produce different alterations of intermuscular coordination. For now, the proposed rehabilitation method can be implemented in the isokinetic exercise condition. The isokinetic movements have several advantages for training of muscle activation—for example, a muscle can be activated at a varying posture during movement, the training is safe because of the limited speed of end-point or a particular joint movement, and co-contraction of agonist and antagonist muscles during force generation can be reduced [23].

### Study limitations

The current study is limited because the study examined intermuscular coordination of isokinetic movements only. Instead of examining the other task constraints such as free movement, constrained movement, or isometric strengthening in addition to isokinetic exercise, the study referred to the intermuscular coordination patterns of the biomechanical conditions described above from the previous literature. The alteration in intermuscular coordination post stroke observed in this study was consistent with that observed for other tasks with external constraints. The variability of participants and analysis methods between the current and the previous studies, however, still limits the interpretation of the effects of the task constraints on intermuscular coordination. To verify the effect of task conditions on intermuscular coordination, a previous study conducted in the multiple sclerosis population [32] recommended a comprehensive study design including various external constraints.

Second, the end-point force and the intermuscular coordination were compared between the groups in a limited manner. The three-dimensional end-point force was measured by the experimental setup KULSIS, but only the maximum magnitude and the direction of the force were compared between stroke and control groups because the detailed quantification of the end-point force was beyond the scope of the current study. In addition, the alteration of intermuscular coordination was not compared among the stroke participants with respect to their motor capability such as an ability to control the end-point force. In the future study, based on the detailed quantification of the end-point force, stroke survivors can be divided into several subgroups depending on the evaluation of the end-point force. Then, muscle synergies can be compared between not only the stroke and control groups, but also the subgroups of the stroke participants. The impact of the abnormal intermuscular coordination post stroke on the ability to control the end-point force will be thoroughly evaluated to investigate the tailored intermuscular coordination-based rehabilitation.

## Conclusion

We examined alterations in intermuscular coordination of people with chronic stroke during isokinetic upper limb movements along constrained end-point trajectories. The composition of most muscle synergies was fairly conserved after a stroke, although alterations in shoulder-related synergies existed because of the coupling of the deltoid muscles. Stroke participants and neurologically intact participants showed different activation coefficients of the synergies. Similar alterations in intermuscular coordination were observed by previous studies that adopted different types of biomechanical task constraints. We hypothesized that the external constraints (such as force support or kinematic constraint) may help people with a stroke use their synergies in a more task-specific manner. Accordingly, we suggest that providing an external constraint such as isokinetic movement can be beneficial for the practice of independent activation of muscle synergies for functional recovery of the upper limb. In addition to the training of activation of synergies, resolving the coupling of the deltoid muscles can be included in the future intermuscular coordination-based training for more effective rehabilitation.

## Data Availability

The datasets used and/or analyzed during the current study are available from the corresponding author on reasonable request.
